# Degradation behavior of porous magnesium alloy scaffold under the low-intensity pulsed ultrasound intervention and their effect on bone defects repair

**DOI:** 10.1093/rb/rbaf011

**Published:** 2025-03-14

**Authors:** Delin Ma, Mingran Zheng, Jun Wang, Yuan Zhang, Qichao Zhao, Zhaotong Sun, Junfei Huang, Wenxiang Li, Shijie Zhu, Liguo Wang, Xiaochao Wu, Shaokang Guan

**Affiliations:** School of Material Science and Engineering, Zhengzhou University, Zhengzhou 450001, China; Henan Key Laboratory of Advanced Light Alloys, Zhengzhou University, Zhengzhou 450002, China; Zhongyuan Critical Metals Laboratory, Zhengzhou University, Zhengzhou 450001, China; School of Material Science and Engineering, Zhengzhou University, Zhengzhou 450001, China; Henan Key Laboratory of Advanced Light Alloys, Zhengzhou University, Zhengzhou 450002, China; Zhongyuan Critical Metals Laboratory, Zhengzhou University, Zhengzhou 450001, China; School of Material Science and Engineering, Zhengzhou University, Zhengzhou 450001, China; Henan Key Laboratory of Advanced Light Alloys, Zhengzhou University, Zhengzhou 450002, China; Zhongyuan Critical Metals Laboratory, Zhengzhou University, Zhengzhou 450001, China; School of Material Science and Engineering, Zhengzhou University, Zhengzhou 450001, China; Henan Key Laboratory of Advanced Light Alloys, Zhengzhou University, Zhengzhou 450002, China; School of Material Science and Engineering, Zhengzhou University, Zhengzhou 450001, China; Henan Key Laboratory of Advanced Light Alloys, Zhengzhou University, Zhengzhou 450002, China; Zhongyuan Critical Metals Laboratory, Zhengzhou University, Zhengzhou 450001, China; School of Material Science and Engineering, Zhengzhou University, Zhengzhou 450001, China; Henan Key Laboratory of Advanced Light Alloys, Zhengzhou University, Zhengzhou 450002, China; Zhongyuan Critical Metals Laboratory, Zhengzhou University, Zhengzhou 450001, China; Ltd Shenzhen Branch, Shimadzu (China) Co., Shenzhen 528042, China; Zhengzhou Orthopedic Hospital, Zhengzhou 450053, China; School of Material Science and Engineering, Zhengzhou University, Zhengzhou 450001, China; Henan Key Laboratory of Advanced Light Alloys, Zhengzhou University, Zhengzhou 450002, China; Zhongyuan Critical Metals Laboratory, Zhengzhou University, Zhengzhou 450001, China; School of Material Science and Engineering, Zhengzhou University, Zhengzhou 450001, China; Henan Key Laboratory of Advanced Light Alloys, Zhengzhou University, Zhengzhou 450002, China; Zhongyuan Critical Metals Laboratory, Zhengzhou University, Zhengzhou 450001, China; School of Material Science and Engineering, Zhengzhou University, Zhengzhou 450001, China; Henan Key Laboratory of Advanced Light Alloys, Zhengzhou University, Zhengzhou 450002, China; Zhongyuan Critical Metals Laboratory, Zhengzhou University, Zhengzhou 450001, China; School of Material Science and Engineering, Zhengzhou University, Zhengzhou 450001, China; Henan Key Laboratory of Advanced Light Alloys, Zhengzhou University, Zhengzhou 450002, China; Zhongyuan Critical Metals Laboratory, Zhengzhou University, Zhengzhou 450001, China

**Keywords:** bone defects, porous magnesium alloy scaffolds, LIPUS, degradation behavior, osteogenesis

## Abstract

Biodegradable porous magnesium alloy (pMg) scaffolds hold significant potential for repair of bone defects owing to favorable mechanical properties and biocompatibility. However, a critical challenge remains in matching the degradation rate of pMg scaffolds with the pace of bone regeneration. Low-intensity pulsed ultrasound (LIPUS) has emerged as a promising therapeutic strategy to enhance bone repair. In this study, femoral bone defects in Sprague–Dawley rats were implanted with pMg scaffolds, and LIPUS was applied to the defect sites post-operatively. This study primarily investigated the degradation behavior of pMg scaffolds *in vivo* experiments, as well as their reparative effects on bone defects under LIPUS intervention. *In vivo* analysis revealed that LIPUS intervention accelerated the degradation of pMg scaffolds by loosening the degradation layer, making it more susceptible to erosion. Concurrently, LIPUS enhanced the accumulation of beneficial calcium and phosphorus compounds on the surface of the pMg scaffolds. Furthermore, the pMg + LIPUS group exhibited enhanced bone formation and mineralization around the degradation site compared to the pMg group alone, attributed to the increasing osteocalcin (OCN) and type I collagen (COL-I) as well as reduction in osteolysis by pMg and LIPUS-induced osteogenesis effect. At the 24-week post-surgery, the hardness value (HV) of regeneration bone in the pMg + LIPUS group had a 15% increase compared to the pMg group and approached the HV of healthy bone. In conclusion, the promotion of bone tissue growth rate under the intervention of LIPUS in conjunction with the degradation rate of pMg scaffolds offers a novel clinical strategy for the repair of bone defects.

## Graphical Abstract

**Graphical Abstract:**
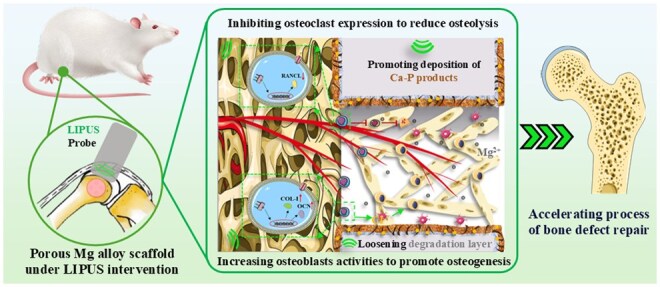

## Introduction

The most common causes of bone defects are congenital diseases, large tumors and severe trauma [1, 2]. The diameter of the bone defects exceeding 50% of the diameter is defined as a large segmental bone defect [3]. In clinical practice, auto-transplantation, allotransplantation and xenotransplantation are the predominant techniques employed for the management of extensive segmental defect disorders [4]. However, these therapeutic treatments come with a few drawbacks, including a lengthy treatment cycle, discomfort, expensive costs and the possibility of rejection [5]. In this context, bone tissue engineering was employed for bone defect repair, a comprehensive therapy technique that combines biomaterial scaffolds and biological components in a number of ways [6]. Besides, the material for the scaffold is the key factor for bone tissue engineering. Currently, commonly used titanium alloys and stainless steels are biologically inert materials and their high mechanical strength is applicable to the load-bearing joint, but their further application is limited by non-degradability and stress shielding effect [7]. Hard and brittle bio-ceramics and low mechanical strength of polymer materials exist the risk of device failure at the early stage of implantation [8]. Therefore, it is vital to employ new materials with superior performance to prepare scaffold [9].

Recently, biodegradable porous magnesium alloy scaffolds have garnered a lot of attention due to good mechanical compatibility and biocompatibility [10–12]. Furthermore, Mg^2+^ released by degradation can promote osteogenesis and accelerate bone defect repair so that bioactive factors are not so necessary for porous magnesium alloy scaffolds [13–15]. Wang *et al*. [16] employed a template replication technique to fabricate Mg–Nd–Zn–Zr and *in vivo* studies demonstrated that these magnesium scaffolds exhibited favorable mechanical properties and promoting the expression of genes associated with osteogenesis. Zhao *et al*. [17] fabricated Mg–1Ca/PCL composite scaffolds using melt blending, and the incorporation of Mg–1Ca significantly enhanced the mechanical strength of PCL and promoted the proliferation, adhesion and osteogenic differentiation of human bone marrow stem cells. However, a critical challenge remains that uncontrolled degradation rate of magnesium alloy scaffold makes it difficult to match the pace of bone healing [18]. Therefore, promoting the bone regeneration to match the degradation rate of scaffold is a current research direction [19]. The commonly used way is to carry drugs on the device surface, but the uncontrolled drug release rate may lead to experimental failure [20]. Adding mechanical stimulation to promote bone growth is a new idea [21, 22].

Low-intensity pulsed ultrasound (LIPUS) has become an effective physical therapy method to promote bone repair because of its non-invasive, effective, safe and convenient operation [23]. Since 1994, the Food and Drug Administration has authorized it for the treatment of newly formed fractures. LIPUS stimulates the expression of several growth factors during fracture repair, such as bone morphogenetic protein (BMP), Osteocalcin (OCN) and vascular endothelial growth factor [24]. LIPUS improves bone repair quality by increasing calcium nodule formation [22]. LIPUS can increase cartilage matrix synthesis and chondrocyte and fibrocyte proliferation and differentiation (type II collagen, glycosaminoglycans, etc.) and improve the maturation and mineralization of new bone [25–27]. Furthermore, LIPUS can be utilized in conjunction with implants to promote bone tissue regeneration. Higuchi *et al*. [28] implanted a porous titanium mesh into rabbit nasal bone defects and demonstrated that applying LIPUS as an adjunctive therapy enhanced the expression of BMP-2, thereby inducing new bone formation. Similarly, Ganzorig *et al*. [29] inserted titanium alloy and stainless-steel implants into the tibiae of rats and revealed that LIPUS stimulation significantly facilitated peri-implant bone formation, thereby improving the initial stability of the implants.

In this study, porous magnesium alloy scaffolds were implanted into the femoral condylar defect model of Sprague–Dawley (SD) rats. Micro-CT, Scanning Electron Microscope Energy and Energy Dispersive Spectrometer (SEM&EDS) and X-ray photoelectron spectroscopy (XPS) were used to observe the degradation behavior of porous magnesium alloy scaffolds under the action of LIPUS. Meanwhile, the synergistic effects of LIPUS and porous magnesium alloy scaffolds on the repair of bone defects were analyzed by imaging, histology and biomechanics.

## Materials and methods

### Preparation of porous magnesium alloy scaffold

**Figure 1.**
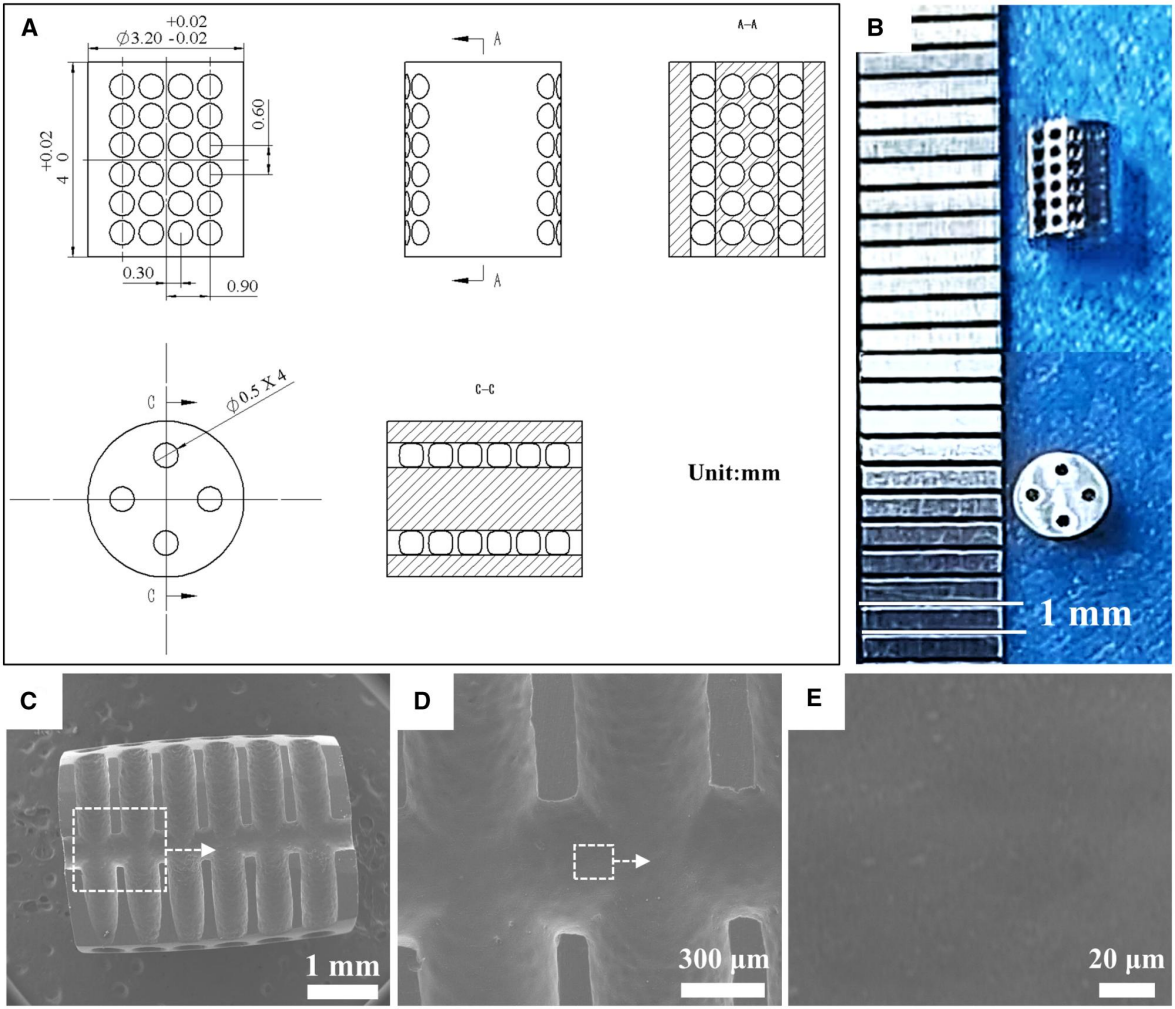
Design of the porous magnesium alloy scaffold (**A**) and the resulting products after electrolytic polishing (**B**). SEM images of the surface morphology of the holes of scaffold (**C**) ×20, (**D**) ×80 and (**E**) ×1000.

Qualified ZE21C magnesium alloy (Mg–2wt%Zn–0.5wt%Y–0.5wt% Nd–0.4wt%Zr) rods were used to manufacture the designed porous scaffolds on a NC EDM machines (D703S-CNC500, China) [30]. All porous magnesium alloy scaffolds (hereafter denoted as pMg) were similar in design and were considered suitable for use in rats. The inner hole size and porosity of the scaffold are 400-500 μm and 50%. The details of the scaffolds are shown in Figure 1A and B. Electrolytic polishing equipment was used to remove machining defects in the holes of scaffold, as shown in Figure 1C–E. Subsequently, all the pMg samples underwent ultrasonic cleaning in absolute ethanol, followed by sterilization using 25 kGy of 60Co radiation, and were subsequently vacuum-packed for preservation.

### Rat femoral condyle bone defects model and LIPUS intervention

**Figure 2.**
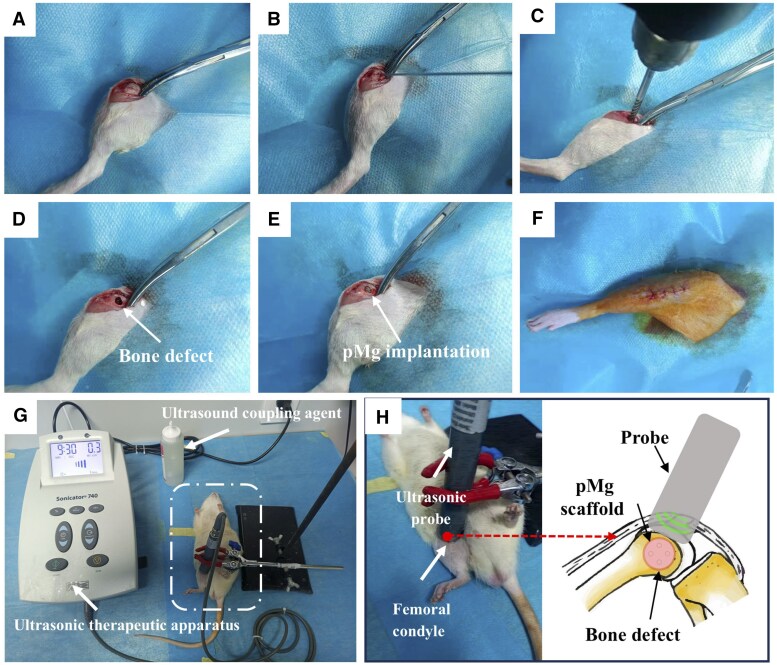
The surgical procedure of the femoral condylar bone defects (**A**)–(**F**) and treatment of bone defects in rats with LIPUS intervention (**G**) and (**H**).

Twenty-four 3-month-old male SD rats weighing 300–310 g (304.7 g on average) were used to establish the femoral condyle bone defects model. The research protocol obtained approval from the Ethics Committee of the Animal Experimental Center at Zhengzhou University, with the reference number SYXK(Yu)2020-0008. The rats were anesthetized via intraperitoneal injection with Pentobarbital (3%, 1 ml/kg). The rats’ right hind legs were shaved, and they were securely fixed on the operating table. Following the placement of a sterile drape over the rats, a 1.5-cm incision was made through the skin and subcutaneous tissue along the medial parapatellar region to expose the femoral condyle (Figure 2A). The hollow drill bit (Φ: 3.2 mm) was used to obtain the bone defect model (3.2 mm × 5 mm) with the help of a traction needle (Φ: 1.5 mm), as shown in Figure 2B–D. Afterward, pMg samples were implanted into the bone defects (Figure 2E), and the muscle and skin wounds were sutured layer by layer with degradable silk thread by turns, and the wound area was wiped with iodophor again (Figure 2F). To avoid wound infection, penicillin sodium was intraperitoneally injected into the rats for three consecutive days post-operatively. The animals were randomly divided into the following two groups: pMg and pMg + LIPUS. Each group was subdivided into four subgroups with implantation times of 4, 8, 12 and 24 weeks.

LIPUS exposure device (Sonicator 740, USA) was used on the pMg groups at 2 weeks post-implantation. The LIPUS intervention treatment process is shown in Figure 2. Simply, the ultrasonic therapeutic probe was positioned over the repaired femoral condyles, utilizing a thin layer of coupling gel for optimal interface (Figure 2G). LIPUS was applied to the bone defect for 10 minutes every day (Figure 2H). The device operated in pulse mode with a frequency of 3 MH, a duty cycle of 10% and an intensity of 30 mW/cm^2^, commonly used parameters in clinical practice [31]. The rats were sacrificed at 2, 4, 8 and 12 weeks post-operatively and the bone samples with implants were placed in paraformaldehyde neutral fixative for follow-up studies.

### X-ray examination

X-ray scanning was conducted on the femoral condyle bone defects of the rats at 4, 8, 12, and 24 weeks post-surgery to assess the location of the scaffolds and to monitor the morphological alterations at the implantation site, encompassing bone formation and the presence of gas generation.

### Micro-computed tomography assessment

Implants and new bone of the femoral condyle samples were analyzed by using a micro-computed tomography (micro-CT) device (SMX-225CT, Shimadzu, Japan). In brief, the samples were placed in the sample table and set the following scanning parameters: 115 kV and 70 μA in the X-ray tube; scanning resolution, 15 μm; and exposure time, 1000 ms. 2D images were obtained and three-dimensional images were obtained by VG-Studio max 3.4 software. The volume of the implant (Φ: 3.2 mm, H: 4.2 mm) was marked as the region of interest (ROI). The new bone threshold interval was set to 400–1200, and the implant threshold interval was set to 1200–1300. The 3D reconstruction was performed of the ROI to calculate the percentages of bone volume (BV)/total volume (TV) (BV/TV), the number of bone trabeculae (Tb. N), the thickness of bone trabeculae (Tb. Th), the separation degree of bone trabeculae (Tb. Sp), the percentages of scaffold degradation volume/original volume (ΔV/V), and degradation rate of the scaffold.

### Porous scaffold surface morphology and composition analysis

After Porous scaffold removal, the surface morphology was observed by scanning electron microscopy (SEM, SU-3500, Hitachi, Japan) and energy dispersive spectrometer element analysis (EDS, EDAX, Element, USA). The non-decalcified tissues containing implants were dehydrated in ethanol (75%, 85%, 90%, 95% and 100%) and xylene at different concentration gradients. Subsequently, the samples were embedded in methyl methacrylate (MMA) for preservation and sectioning. The embedded samples underwent grinding and polishing procedures to achieve a smooth surface. A thin layer of gold was then sputter-coated onto the surface. The morphological features and the distribution of chemical elements within the cross-sectional area were meticulously observed and analyzed by using SEM&EDS.

X-ray photoelectron spectroscopy (XPS, AXIS Supra, Shimadzu, Japan) was used to characterize the electrolytic polishing treatment and *in vivo* degradation products with the following test parameters: an Al Kα monochromatic ray source at 140 W. Qualitative and semi-quantitative analyses were performed on the *in vivo* degradation product compositions and elements of the scaffolds.

### Histological analysis

The histological sections of the repaired femoral condyles were stained. The femur-containing implants were taken from the killed rats, and the femur was placed in a 4% paraformaldehyde solution for fixation for 24 hours. A 0.2-mm thick blade was used to cut the femur into two halves along the sagittal plane of the femoral condyle. One-half of the sample was designated for decalcification and subsequent tissue staining, while the other half was intended for embedding in a hard tissue medium to facilitate the observation and analysis of the scaffold’s degradation behavior. Rapid decalcification was performed with Ethylenediaminetetraacetic acid (EDTA) decalcification solution (Servicebio, China), followed by dehydration with graded ethanol series (Sinopharm Chemical Reagent, China), cleaning with xylene, and embedding with paraffin for histological examination. Slice continuously along the sagittal plane of the femoral condyle into 5-μm thin sections, and then hematoxylin-eosin (H&E), Goldner’s and Von Gieson (VG) staining was performed for the assessment of the new bone directional growth state at the bone defects.

### Immunohistochemistry analysis

Immunohistochemical staining was performed on two groups of 12 weeks and 24 weeks paraffin embedded tissues. Briefly, the paraffin sections were first dewaxed and dehydrated, rinsed three times with 3% H_2_O_2_ solution at room temperature for 3 min each time, washed three times with 5% Phosphate buffered saline (PBS) solution, and finally closed with 5%. The sections were incubated in Bovine Serum Albumin (BSA) solution at room temperature for a duration of 20 minutes. Following this, they were exposed to primary antibodies (RANCL, TRAP, OCN and COLI) at 4°C overnight. Subsequently, the sections were thoroughly washed with PBS solution three times. After washing, the corresponding secondary antibody solution was added. The sections were then incubated at room temperature for 60 minutes. Following this incubation period, a freshly prepared DAB-H_2_O_2_ chromogenic solution was introduced to the sections. Finally, the sections were stained with hematoxylin and mounted onto slides for encapsulation. Following this, the stained sections were analyzed under a fluorescence inverted microscope. Images were captured to document the observations.

### Bone tissue hardness testing

Thin slices of 100-μm thickness were obtained from the MMA-embedded hard tissues. A microhardness tester (HV-1000A, China) was employed to measure the hardness vulue (HV) of these bone slices. During the measurement, a loading weight of 25 g was applied for a duration of 10 seconds to create indentation marks. The diagonal length of these indentation marks was then utilized by the built-in software to calculate the HV value.

### Statistical analysis

The SPSS 22.0 software (SPSS Inc, Chicago, USA) was used to transform and statistically analyze the data. The significant difference in the data was determined by the *t*-test, and each group of measured values plotted in the figure was expressed by mean ± SD. *P* < 0.05 is considered indicative of statistical significance.

## Results

### X-ray imaging analysis

**Figure 3.**
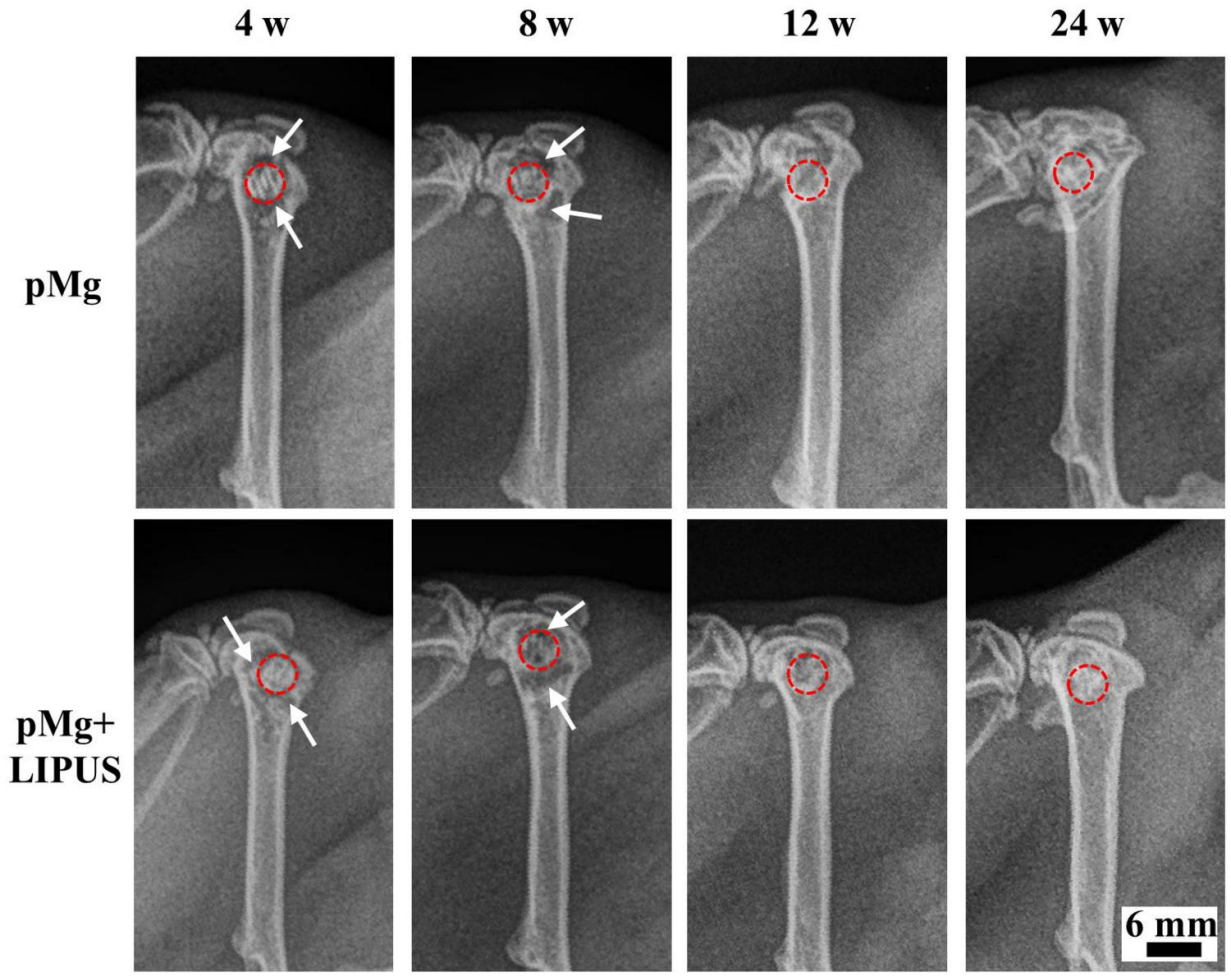
Representative radiographic images at 4, 8, 12 and 24 weeks after implantation. Arrows: low-density shadows, Rings: scaffold outline.

X-ray scanning was shown in Figure 3. At 4 weeks after operation, some low-density shadows appeared around the scaffolds in two groups. In comparison to pMg group, the contour of scaffold in pMg + LIPUS group became unclear. At 8 weeks after operation, the shadow area surround the femoral condyles decreased in two groups. At 12 weeks after operation, there was not gas shadow and contour of scaffold in two groups. At 24 weeks post-surgery, the gray density of the implantation site increased, and the pMg + LIPUS group was denser than the pMg group. During the whole experiment, there was no scaffold dislocation in both groups.

### *In vivo* degradation behavior of porous magnesium alloy scaffold

**Figure 4.**
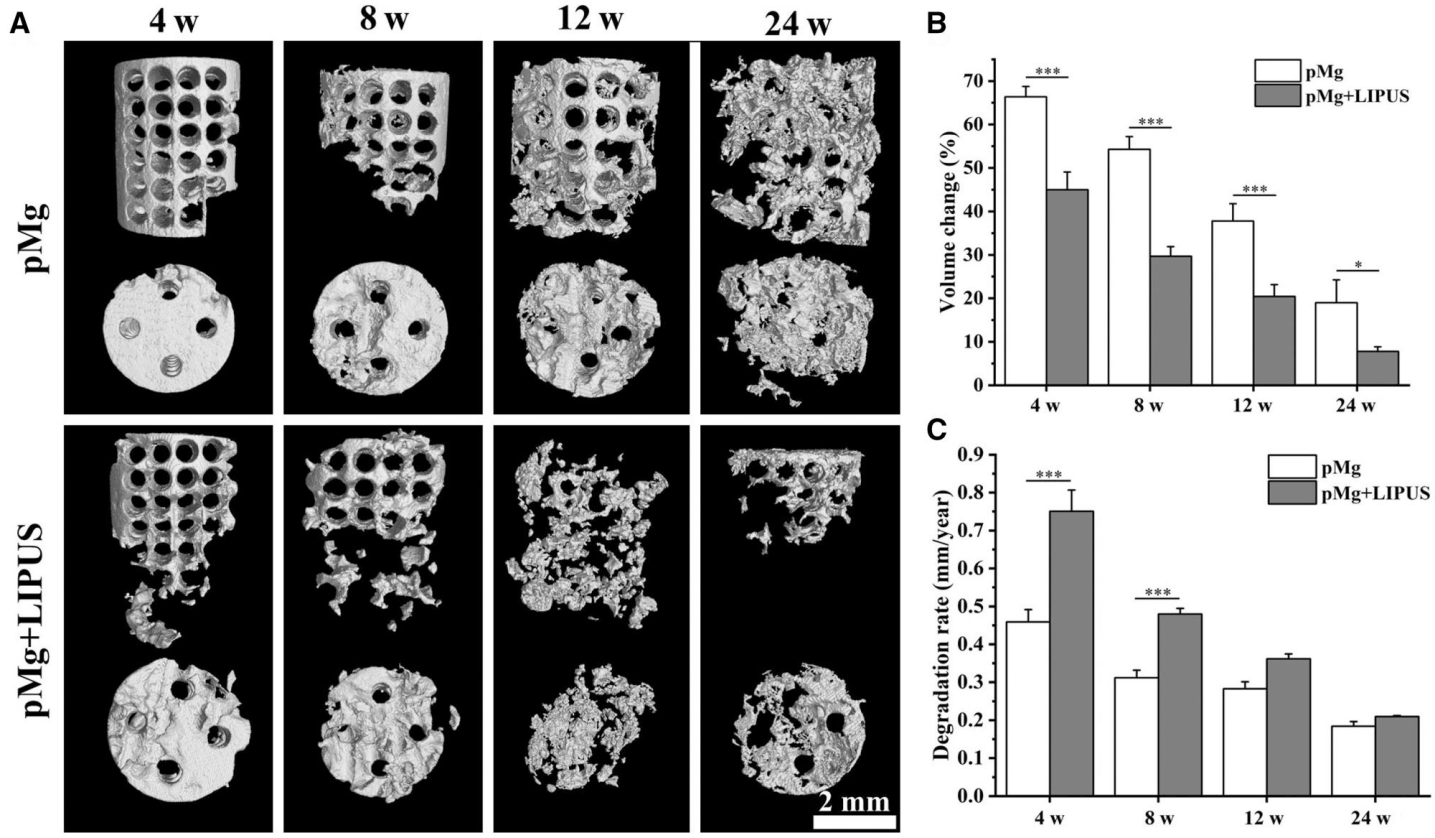
(**A**) 3D Micro-CT images of the implanted porous magnesium alloy scaffold. *In vivo* (**B**) volume change and (**C**) degradation rate of porous magnesium alloy scaffold at 4, 8, 12 and 24 weeks post-implantation.

Based on the micro-CT analysis of the three-dimensional morphology of the pMg scaffolds, observations revealed a gradual degradation of the scaffolds in both groups over time. Furthermore, the degree of scaffold degradation in the pMg + LIPUS group was observed to be greater than that in the pMg group at each observation point (Figure 4A). At 4 weeks post-implantation, the scaffolds near damaged cortical bone in the pMg + LIPUS group exhibited more obvious local degradation. The scaffolds in the pMg + LIPUS group had an average degradation rate of 0.75 mm/year and approximately 45% of the original volume (Figure 4B and C). At 8 weeks post-implantation, both groups further degraded, but some complete pore structures remained. At 12 weeks post-surgery, the porous structure of pMg group remained relatively complete, while the porous structure of the pMg + LIPUS group could not be observed, and the remaining volume was 20.43% of the original volume. At 24 weeks post-implantation, the degradation rate of both experimental groups remained around 0.2 mm/year. The remaining volume of scaffolds in the pMg group was 18.97% and the pMg + LIPUS group was about 7.8%.

**Figure 5.**
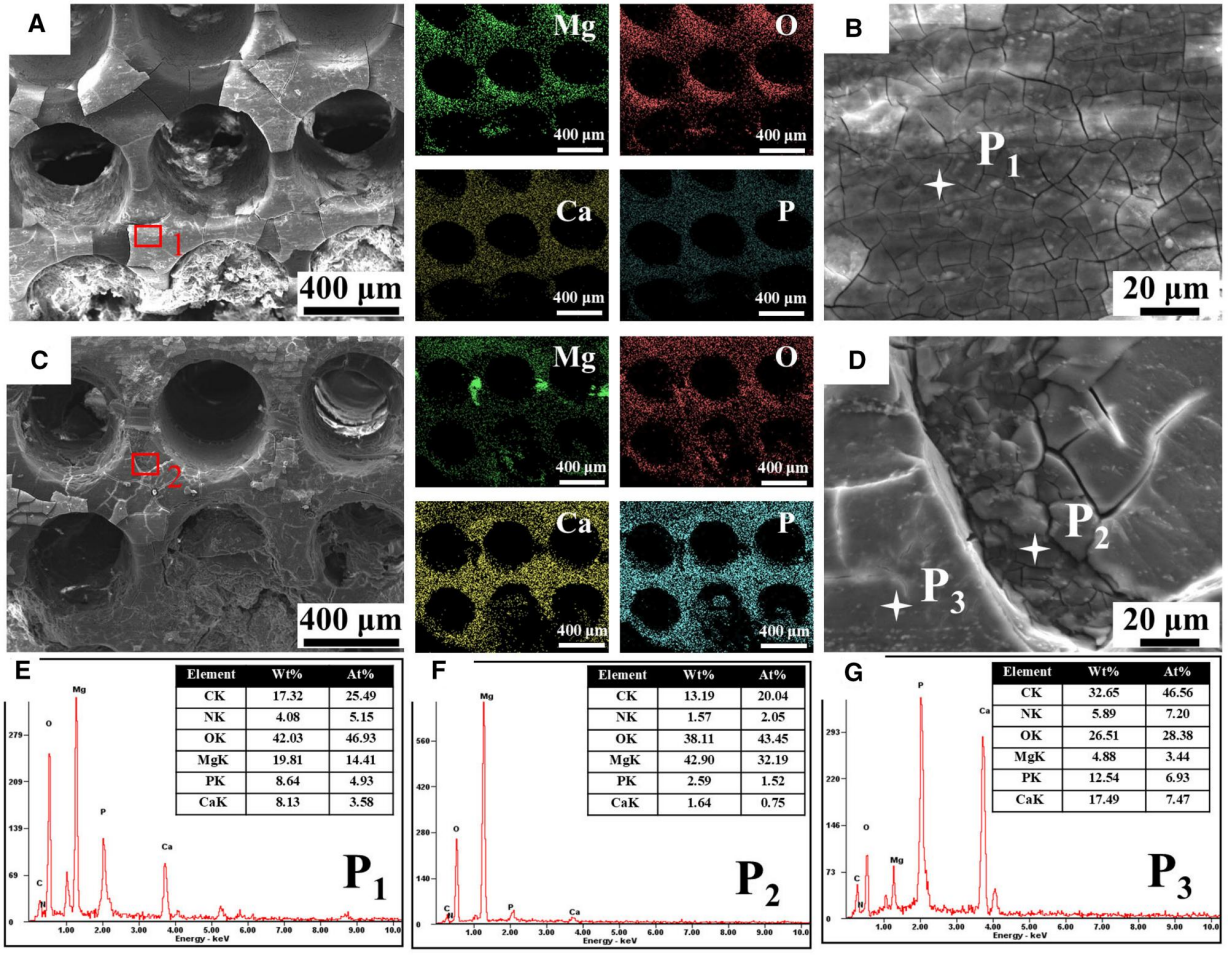
SEM Morphologies of the pMg group (**A** and **B**) and pMg+LIPUS group (**C** and **D**) after 4 weeks post-implantation. EDS data for P_1_ (**E**), P_2_ (**F**) and P_3_ (**G**).

Figure 5 showed representative SEM morphologies of two groups at 4 weeks post-surgery. It can be observed that tissue was present in the pores of two groups (Figure 5A and C). The concentration of magnesium (Mg) and oxygen (O) was found to be higher in the pMg group than in the pMg + LIPUS group. In contrast, the surface of the pMg + LIPUS group exhibited increased levels of Ca and P elements compared to the pMg group. Microscopically, the surface of pMg was in a dry riverbed corrosion state (Figure 5B) and the surface elements were mainly O and Mg (Figure 5B and E). For pMg + LIPUS group, some regions had extremely high content of Mg elements, and the microstructure was in a big fracture (Figure 5D and F). In contrast, higher Ca and P content was around cracks (Figure 5G).

**Figure 6.**
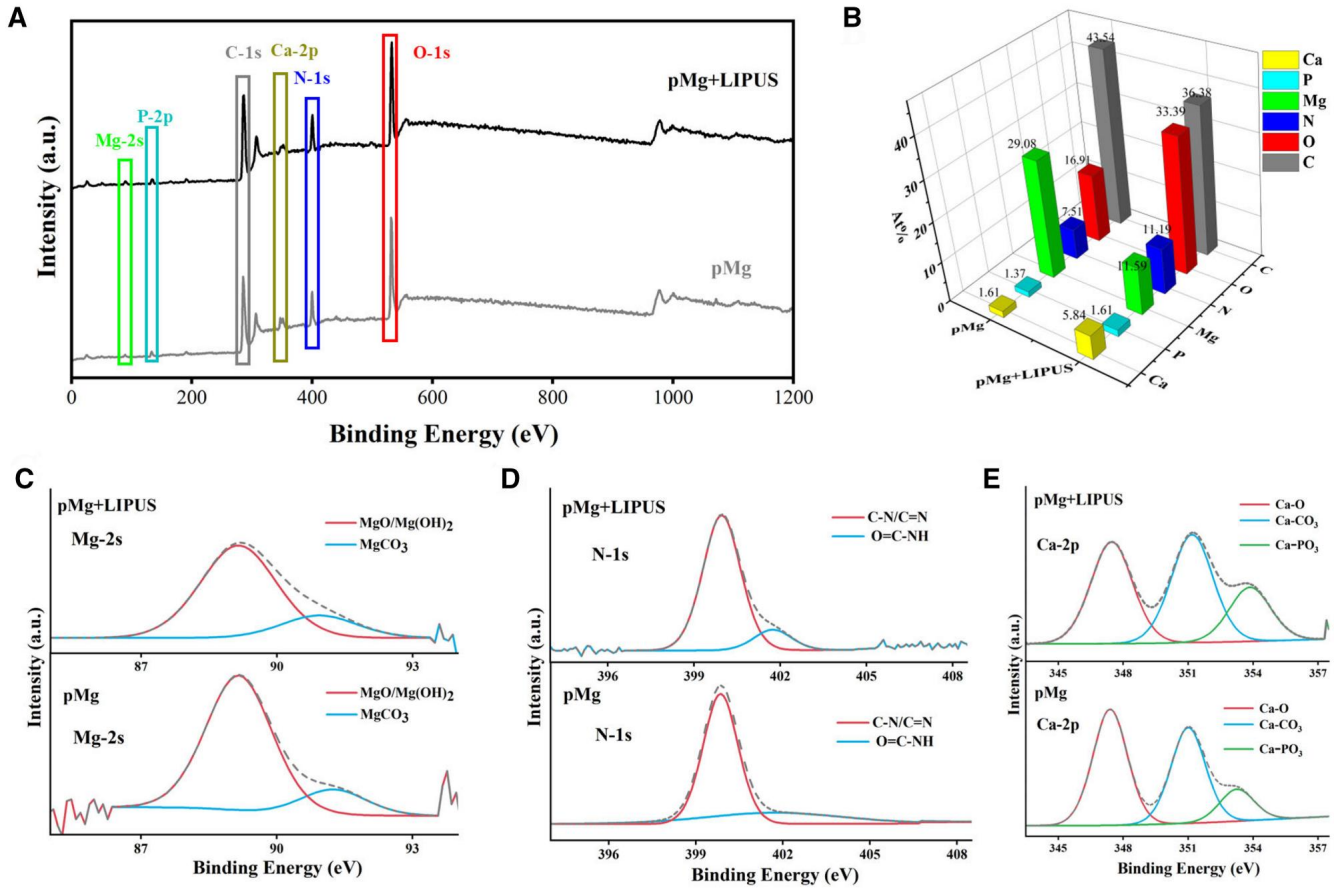
XPS spectra of degradation products of different specimens (**A**) XPS broad spectrum of degradation products; (**B**) XPS elemental content of degradation products; (**C**) Mg-2s narrow spectrum; (**D**) N-1s narrow spectrum; (**E**) Ca-2p narrow spectrum.

Figure 6 presented the results of XPS analysis of the scaffolds’ degradation products under the action of LIPUS. Figure 6A and B showed that distinct C, N, O, Mg, P and Ca peaks can be detected in both pMg and pMg + LIPUS groups. From the element content of surface products, the Mg element content in the pMg + LIPUS group was lower than the pMg group. In contrast, the Ca and N as well as C element in the pMg + LIPUS group were higher than in the pMg group. A distinct absorption peak was detected at 89.17 eV indicating the presence of Mg (OH)_2_, and a weak absorption peak representing MgCO_3_ was also detected at 91.25 eV (Figure 6C) [32]. Besides, the intensity and content of the absorption peaks in the pMg group were higher than the pMg + LIPUS group, suggesting that more of the degradation products on the surface are Mg-based degradation products. Figure 6D showed 399.85 eV and 401.72 eV of the two fitted peaks of N-1s in the degradation products. It connected with the presence of for C–N bonds and for C=O–N–H bonds in organic compounds [33, 34]. Furthermore, higher absorption peak intensities of C=O–N–H in the pMg + LIPUS group suggesting more protein-related products on its surface. Figure 6E showed that the 347.5 eV (Ca-2p3/2), 351 eV (Ca-2p1/2) and 353.25 eV of Ca-2p high resolution spectra can be attributed to calcium in oxide, carboxide or phosphate [35, 36]. The pMg + LIPUS had higher absorption peak intensities and contents of Ca-CO_3_ and Ca-PO_3_, suggesting that the loading of LIPUS promoted the presence of Ca-based degradation products on the surface of the scaffolds.

**Figure 7.**
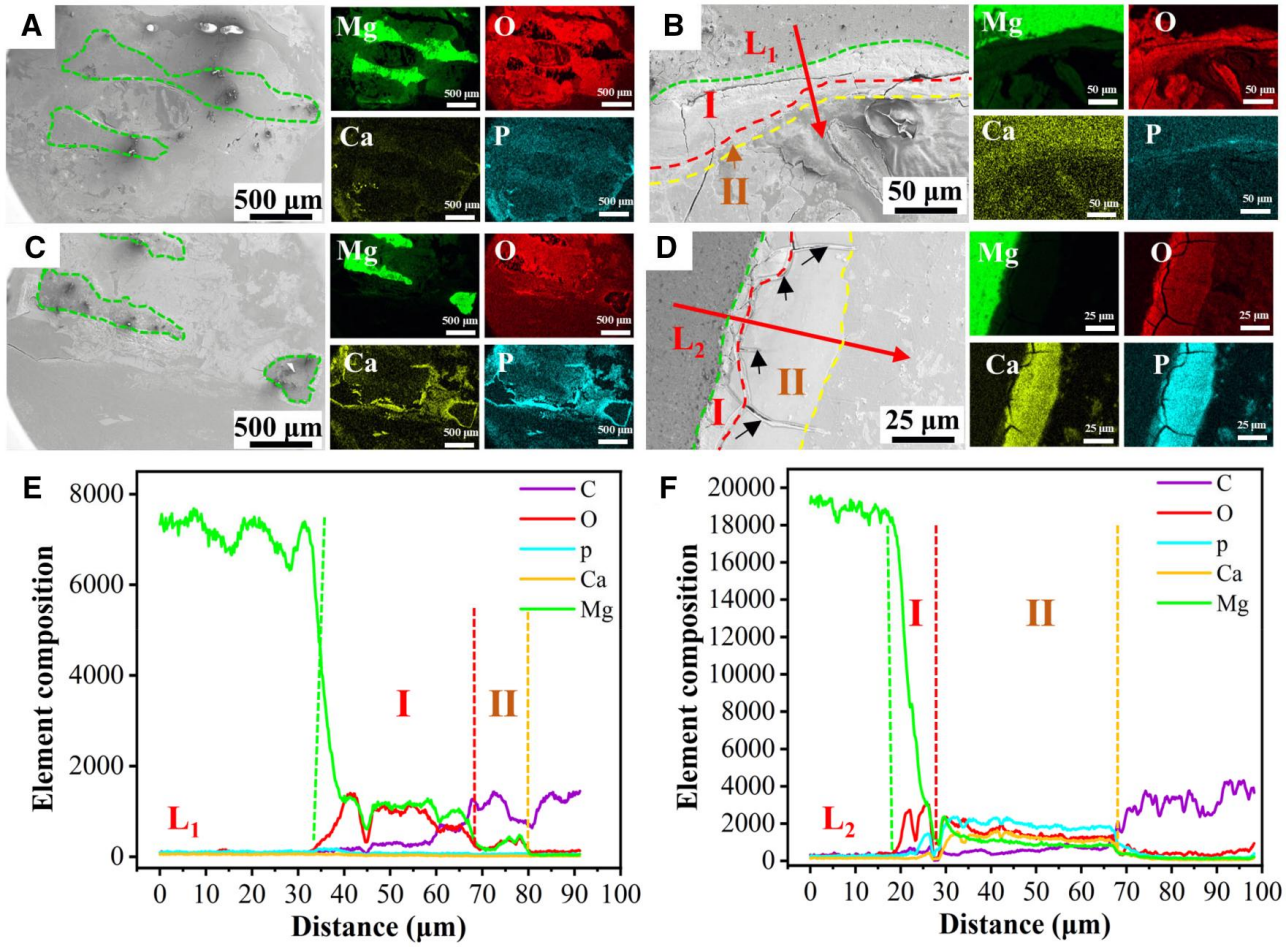
SEM images and surface scan element distribution of the pMg inner hole at 4 weeks post-implantation (**A**) ×50 and (**B**) ×500. SEM images and surface scan element distribution of the pMg+LIPUS inner hole 4 weeks post-implantation (**C**) ×50 and (**D**) ×1000. (**E**) L_1_ line scan element distribution. (**F**) L_2_ line scan element distribution. Green, red and yellow dashed line: corrosion product delamination interface; black arrows: cracks.

The pMg + LIPUS group exhibited higher levels of Ca and P elements compared to the pMg group, while demonstrating lower content of Mg and O elements (Figure 7A and C). From the cross-section morphologyies, pMg group had a thick layer of MgO/Mg(OH)_2_ layer in I region and MgCO_3_ in II region (Figure 7B and E). In contrast, the thickness of I region of the pMg + LIPUS group was lower than the pMg group, and the II region structure was rich in calcium and phosphorus products. Furthermore, I and II regions were loose and had many cracks (Figure 7D and F). The results indicated that the impact of LIPUS on the corrosion layer was mainly reflected in promoting the deposition of Ca and P products on the one hand and made the corrosion layer loose to be eroded easily in another hand.

### Bone healing around porous magnesium alloy scaffold

**Figure 8.**
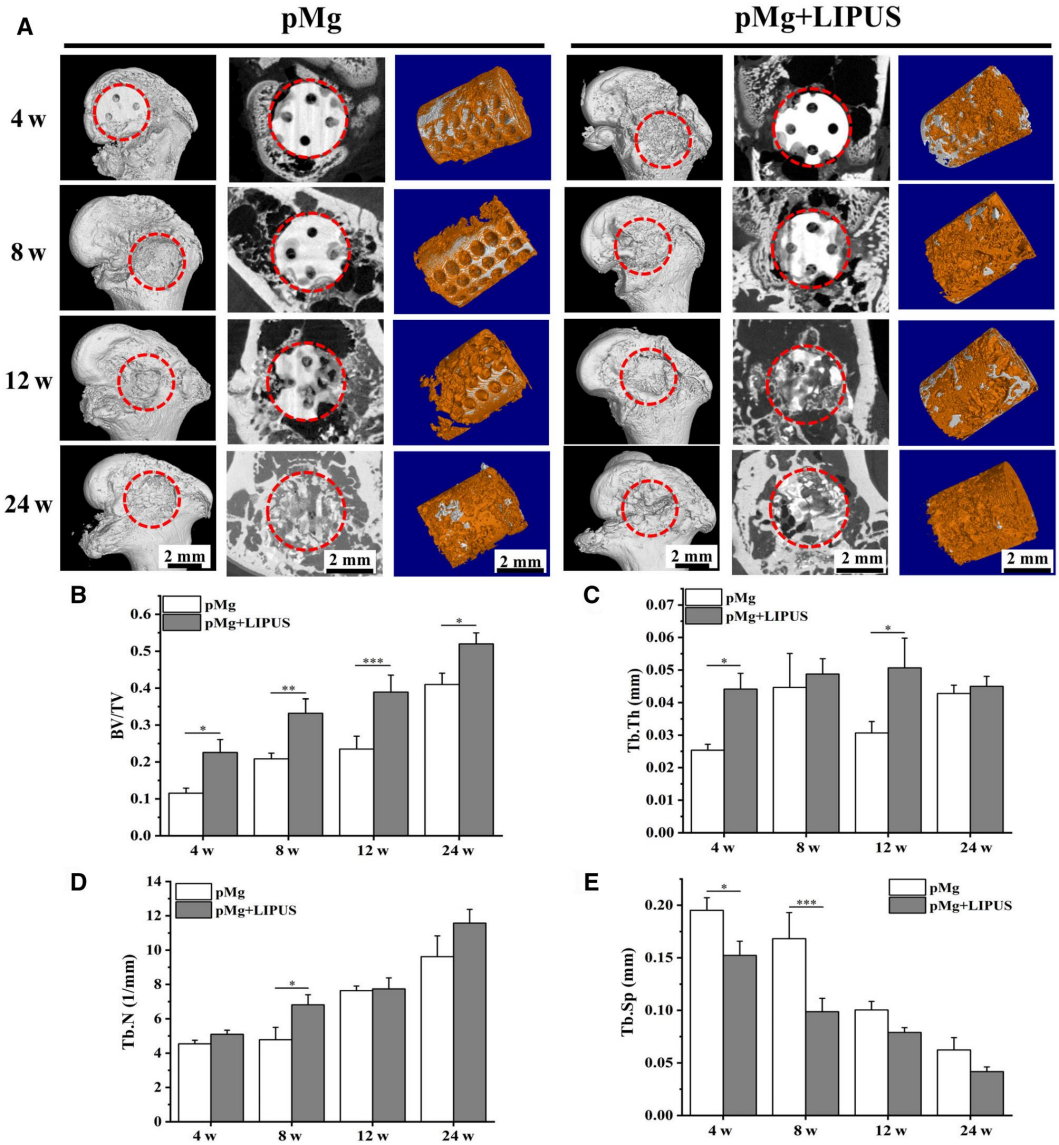
Micro-CT analysis of the scaffold and bone mass around the scaffold. (**A**) Reconstructed 3D models and representative 2D micro-CT images of femoral condylar bone defect and the remaining scaffolds and newly formed bone around the scaffolds at 4, 8, 12 and 24 weeks after post-surgery. Rings indicate the region of interest. The parameters of bone trabecular structure BV/TV (**B**), Tb. Th (**C**), Tb. N (**D**) and Tb. Sp (**E**) around the scaffold within 24 weeks after the operation (*n* = 3). Data are presented as the mean ± SD. *P < 0.05, **P < 0.01, ***P < 0.001.

Three-dimensional micro-CT was utilized for the reconstruction of femoral condyle defects in rats from both groups. At 4 weeks post-implantation, the regenerated tissue had covered the scaffold in the pMg + LIPUS group, while the scaffold was still exposed to the outside in the pMg group. As the implantation time went on, the regenerated bone tissue gradually filled with defects. At 12 weeks post-surgery, the surface cortical bone in the pMg + LIPUS group basically healed (Figure 8A). The reconstructed images obtained at 4, 8, 12 and 24 weeks post-implantation depicted the location of the scaffolds and the status of new bone formation. At 4 weeks post-implantation, both groups exhibited a minor amount of new bone formation surrounding the scaffolds. In the pMg group, the degraded portion of the scaffold was not adequately filled with newly formed bone at 8 and 12 weeks post-surgery. Conversely, in the pMg + LIPUS group, significantly more new bone tissue was observed surrounding the degraded part of the scaffold at these time points. At 24 weeks post-surgery, an increased number of trabecular structures were observed at the surround of the scaffold. The measurement results of BV/TV, Tb. N, Tb. Th and Tb. Sp were shown in Figure 8B–E. During the 24-week implantation period, the pMg + LIPUS group exhibited significantly higher BV/TV and Tb.N compared to the pMg group, while demonstrating a lower Tb.Sp than the pMg group.

### Histological analysis

**Figure 9.**
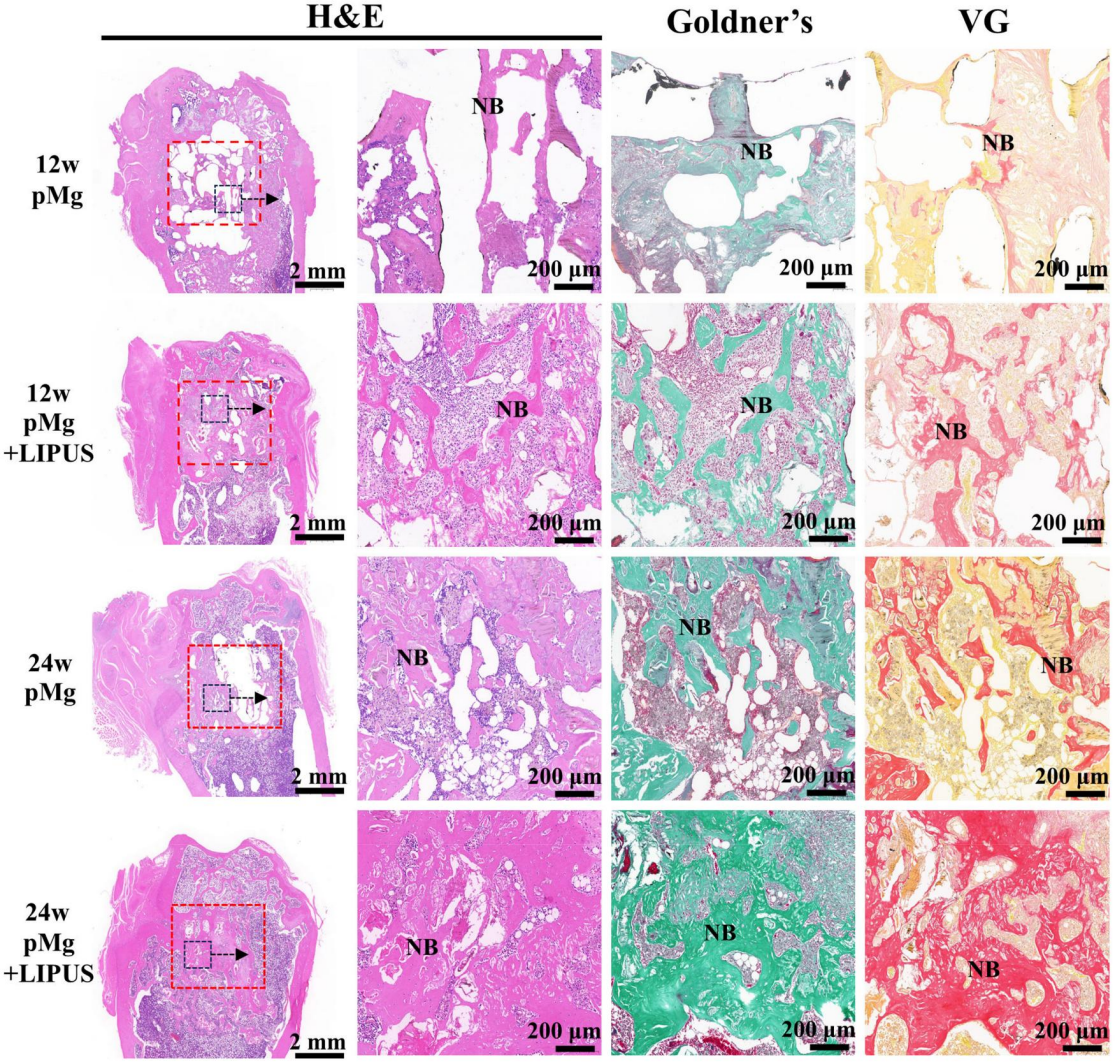
H&E staining, Goldner’s and VG staining results of the femoral condylar defect after scaffold implantation in SD rats for 12 and 24 weeks. Boxes: scaffold insertion location. NB: Newly formed bone.

Figure 9 displayed the results of HE staining. At 12 weeks after surgery, a small amount of new bone trabecula grew along with the scaffold foramen to interconnect in the pMg group, while the more thicker bone trabecula almost filled with the defect site in the pMg + LIPUS group. At 24 weeks after surgery, while substantial newly formed bone was observed in the pMg group, some areas of bone defect remained unrepaired. In comparison, the pMg + LIPUS group exhibited a greater amount of well-organized trabecular bone filling the defect area compared to the 12-week after surgery. Goldner’s staining and VG staining results showed that cartilage formation was observed in the pMg group 12 weeks after surgery. In contrast, the new bone in the pMg + LIPUS group has a higher maturity. At 24 weeks after surgery, the pMg + LIPUS group demonstrated a higher degree of bone mineralization compared to the pMg group, with larger bone tissue, thicker trabecular bone and smaller mineralized bone spacing.

### Immunohistochemistry

**Figure 10.**
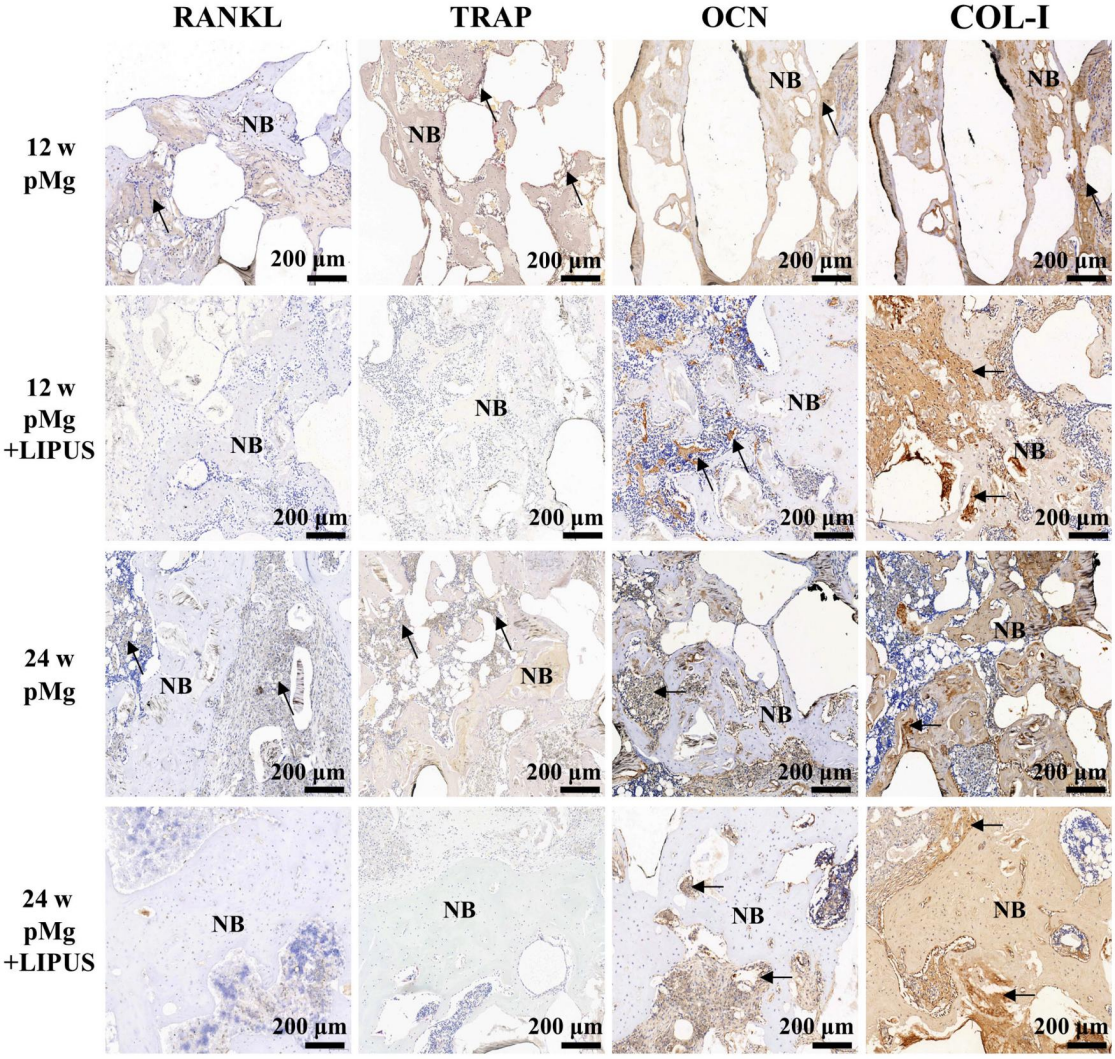
Immunohistochemical staining. RANKL, TRAP, OCN and COL-I protein was expressed at 12 and 24 weeks. Arrows: positive expression. NB: Newly formed bone.

The expression of related factors, including RANKL, TRAP, OCN and COL-I, in bone remodeling was analyzed at 12 and 24 weeks post-operation by immunohistochemical staining (Figure 10). The results showed that osteoclast-associated precursor RANKL with the marker TRAP was strongly expressed in the pMg group at 12 weeks post-operatively. Furthermore, pMg group at 24 weeks post-operation still had relatively higher expression than the pMg + LIPUS group. The OCN was related to osteogenesis and pMg + LIPUS group had higher expression than the pMg group at 12  and 24 weeks post-operatively. COL-I expression promoted angiogenesis and reflected the quality of bone tissue formation in bone defects. The pMg group had a low expression of COL-I. For the pMg + LIPUS group, strong expression was around bone tissue at 12 weeks and within bone tissue at 24 weeks post-operatively.

### Bone tissue hardness results

**Figure 11.**
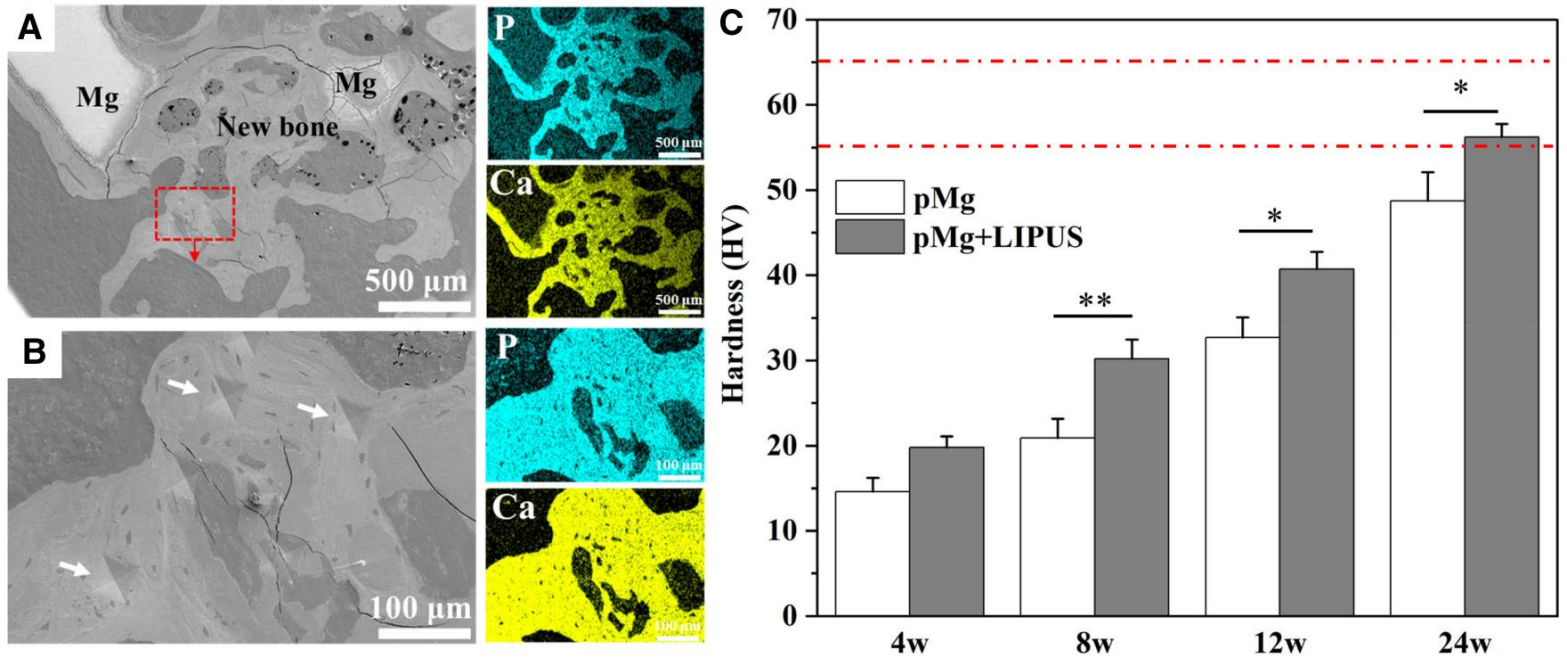
Morphology and face scan of newly formed bone at bone defects (**A**) ×100, (**B**) ×500, (**C**) the hardness value (HV) of the pMg and pMg+LIPUS group. Lines: mechanical values of healthy bone (55–65). Data are presented as the mean ± SD. *P < 0.05, **P < 0.01.

The HV results were shown in Figure 11. The HV location was the red box location of Figure 11A. As shown in Figure 11B, the white arrow showed the location of the indentation left by the HV experiment. Figure 11C showed the trend of HV variation obtained from the calculation of indentation for different surgery times. In all groups, a gradual increase in the hardness of newly formed bone was observed over time. Notably, the hardness value (HV) of newly formed bone in the pMg + LIPUS group was higher than that in the pMg group at each time point. In particular, At the 24-week period, the hardness value (HV) of the pMg + LIPUS group reached 56, representing a 15% increase compared to the pMg group (*P < 0.05) and approaching the hardness level of normal bone.

## Discussion

In this study, the bone defect model in SD rats was used to investigate the degradation behavior of porous magnesium alloy scaffolds under LIPUS intervention and to evaluate their combined effects on bone defect repair. Imaging, histological and biomechanical results revealed that LIPUS accelerated degradation of porous magnesium alloy scaffolds, and their synergistic repair exhibited superior osteogenic properties than using porous magnesium alloy scaffold along.

In this study, low-density shadows were observed in the femoral condylar and external cavities of both scaffold groups at 4 weeks post-implantation (Figure 3). This phenomenon may be attributed to hydrogen gas released during the rapid degradation of the scaffolds in the early post-operative period [37]. This observation is consistent with the findings of Chen *et al.* [38], who reported gas production around magnesium alloy implants at four weeks post-implantation. As time progressed, the shadow gradually diminished after 8 weeks and completely disappeared at 12 weeks post-implantation, which was consistent with the gradual decline in degradation rate shown in Figure 6C. Furthermore, gas production did not interface bone tissue growth around porous magnesium alloy scaffolds (Figures 9 and 10). These results were similar to the research of Antoniac *et al*. [39] implanted magnesium alloy blocks in rabbits to observe the gas effect, and reported that gas did not cause pathological structural changes in muscle or bone in animal models. Notably, the Mg + LIPUS group had similar shadows to the Mg group in the first 8 weeks and did not have large gas shadows due to excessive degradation. That might be that LIPUS caused fluid flow around the tissues as well as the formation of vascularization, accelerating the transit of generated gases [40].

In this study, the degradation rate of the pMg under the LIPUS intervention was faster than that of pMg in implantation surgery (Figure 4). From the surface and cross-section of scaffolds in the pMg + LIPUS group, the corrosion layer of the scaffold had more cracks so that the aggressive ions were more likely to come into contact with the matrix (Figures 5 and 7). This can be attributed to the fact that ultrasound as a mechanical wave produces a cavitation effect [41, 42]. Micromechanical flows and vibrations due to cavitation effects can crack the degradation product layer. As the time of implantation increased, pMg + LIPUS group was reduced to a similar rate to that of pMg. It further suggested that the cavitation response of LIUPS has a strong influence on the pre-implantation degradation product layer. Furthermore, LIPUS enhanced the metabolism of surrounding tissues, thereby accelerating the degradation of magnesium alloy scaffolds [22, 43]. In addition, LIPUS accelerated degradation while promoting the deposition of calcium and phosphorus products on the surface of magnesium alloy scaffolds. The reason for the facilitated deposition of Ca and P products is the local alkaline microenvironment resulting from the ongoing degradation of Mg alloys [44]. LIPUS also promoted the adsorption of proteinaceous substances to some extent (Figure 6). This is due to the fact that proteinaceous substances have a certain pH buffering effect [45].

The effect of LIPUS in collaboration with porous magnesium alloy scaffold on the repair of femoral condylar defect was observed in this study. Micro-CT imaging results showed that compared with the pMg group, pMg + LIIPUS group had higher bone volume fraction and better bone trabecular parameters at each observation point (Figure 8). Liu *et al.* [46] also found that LIPUS increased BV/TV, Tb.Th, Tb.N and significantly reduced Tb.Sp at bilateral tibial bone defects in rats. In addition, more new bone grew along the internal pore of the scaffold in the pMg + LIIPUS group from the 2D images and 3D reconstructed models (Figure 8A), which suggested new bone growth rate matched the degradation rate better under the LIPUS intervention [47]. Furthermore, histological staining showed that the number of bone cells increased under the synergistic effect, new bone mineralization inside the bone defect was transformed into bone trabecular structure at 12 weeks post-surgery. The maturity of new bone under LIPUS was higher and the new bone could extend outwards and connect with each other to form a mesh bone trabecular structure at 24 weeks post-surgery (Figure 9). Besides, the synergistic effect observed in the pMg + LIPUS group led to improved quality of new bone mineralization, and the bone hardness at 24 weeks basically reached the normal bone level (Figure 11).

**Figure 12.**
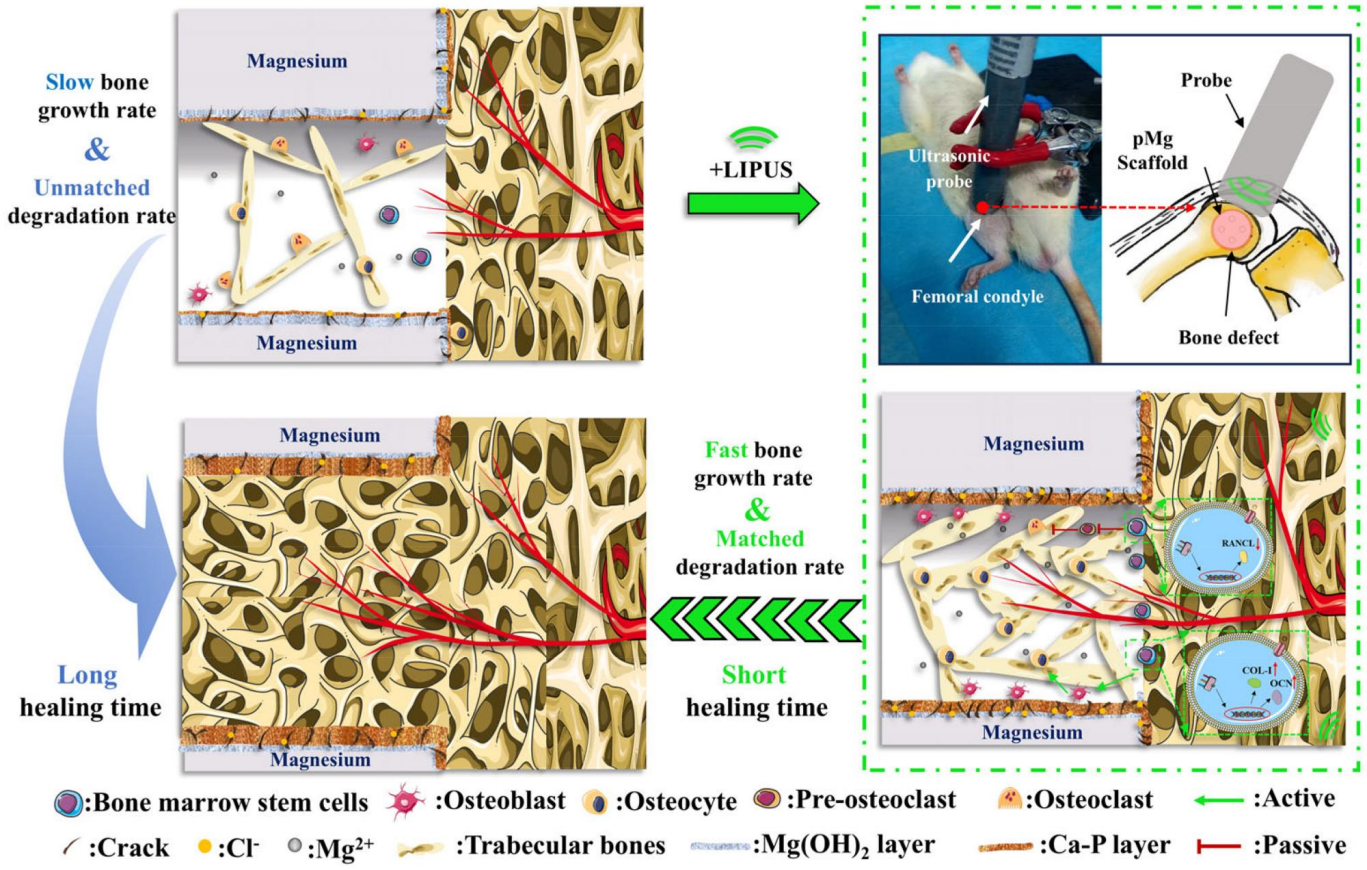
Schematic diagram of mechanisms behind the repair effects of porous magnesium alloy scaffold under the LIPUS intervention in the bone healing.

The osteopromotive effects of pMg scaffolds in bone defects under the LIUPS intervention may be ascribed to three possible reasons, as shown in Figure 12. Firstly, Mg are known as beneficial elements for bone healing. A number of studies have found the mechanism that Mg^2 + ^ released by magnesium alloy degradation promotes bone injury repair [48–50]. Large amounts of Mg^2 + ^ released by fast degradation diffused rapidly under LIUPS intervention to promote the healing process. Concomitantly, LIPUS promotes bone formation by increasing the activity of osteoblasts. Thus, more OCN and COL-I form osteoblasts can be secreted to mobilize bone marrow stem cells toward the defect sites and to promote angiogenesis, thus accelerating bone healing [51, 52]. In addition, pMg + LIPUS group had osteoclasts weakly expressed from the TRAP staining results (Figure 10). This was due to that LIPUS was able to inhibit osteoclast expression further reducing osteolysis by suppressing the expression of the differentiation factor RANKL [53]. Thus, porous magnesium alloy scaffolds under LIPUS intervention showed a better effect of promoting bone growth, which may be attributed to scaffold structure providing direction for new bone interconnection, magnesium ions generated by scaffold degradation and the beneficial effect of LIPUS shortening the bone healing cycle.

However, there are some limitations in the present study. To ensure the single effect of LIPUS on magnesium alloy scaffolds, no coating was prepared on the scaffolds. In this study, it was found that the scaffold under the action of LIPUS had low-density gas shadow and severe degradation before 12 weeks post-operation. In the future, coatings on the scaffold would be considered to avoid excessive degradation in the early stage. In addition, the defect of femoral condyle bone area in rats was small. Future research endeavors may consider conducting experiments on larger animal models to further validate the efficacy of LIPUS in conjunction with porous magnesium alloy scaffolds for the repair of bone defects.

## Conclusion

This study investigated the degradation and osteogenic properties of porous magnesium alloy scaffolds with LIPUS intervention in SD rats’ bone defect model. Results showed LIPUS accelerated scaffold degradation while promoting the deposition of calcium and phosphorus products. Owing to the increasing OCN and COL-I as well as reducing osteolysis by pMg and LIPUS-induced synergistic osteogenesis effect, the pMg + LIPUS group led to improved quality of new bone mineralization, and the bone hardness at 24 weeks basically reached the healthy bone level. LIPUS enhancing osteogenic properties to match the degradation rate of porous magnesium alloy scaffolds provides a new clinical strategy for the repair of bone defects.
