# Correction to: Degradation behavior of porous magnesium alloy scaffold under the low-intensity pulsed ultrasound intervention and their effect on bone defects repair

**DOI:** 10.1093/rb/rbaf055

**Published:** 2025-06-17

This is a correction to: Delin Ma, Mingran Zheng, Jun Wang, Yuan Zhang, Qichao Zhao, Zhaotong Sun, Junfei Huang, Wenxiang Li, Shijie Zhu, Liguo Wang, Xiaochao Wu, Shaokang Guan, Degradation behavior of porous magnesium alloy scaffold under the low-intensity pulsed ultrasound intervention and their effect on bone defects repair, *Regenerative Biomaterials*, Volume 12, 2025, https://doi.org/10.1093/rb/rbaf011

The following changes have been made to the originally published manuscript. In the mark-up of the author list in the PDF, the missing diactritic obelisk (“†”) has been added to those authors who contributed equally to the work.

